# Oncostatin M Protects Rod and Cone Photoreceptors and Promotes Regeneration of Cone Outer Segment in a Rat Model of Retinal Degeneration

**DOI:** 10.1371/journal.pone.0018282

**Published:** 2011-03-30

**Authors:** Xin Xia, Yiwen Li, Deqiang Huang, Zhengying Wang, Lingyu Luo, Ying Song, Lian Zhao, Rong Wen

**Affiliations:** 1 Bascom Palmer Eye Institute, Miller School of Medicine, University of Miami, Miami, Florida, United States of America; 2 Department of Ophthalmology, Shanghai First People's Hospital, School of Medicine, Shanghai, Shanghai Jiaotong University, People's Republic of China; 3 Shanghai Key Laboratory for Ocular Fundus Diseases, Shanghai, People's Republic of China; 4 Department of Ophthalmology, School of Medicine, University of Pennsylvania, Philadelphia, Pennsylvania, United States of America; 5 Neuroscience Program, Miller School of Medicine, University of Miami, Miami, Florida, United States of America; Emory University, United States of America

## Abstract

Retinitis pigmentosa (RP) is a group of photoreceptor degenerative disorders that lead to loss of vision. Typically, rod photoreceptors degenerate first, resulting in loss of night and peripheral vision. Secondary cone degeneration eventually affects central vision, leading to total blindness. Previous studies have shown that photoreceptors could be protected from degeneration by exogenous neurotrophic factors, including ciliary neurotrophic factor (CNTF), a member of the IL-6 family of cytokines. Using a transgenic rat model of retinal degeneration (the S334-ter rat), we investigated the effects of Oncostatin M (OSM), another member of the IL-6 family of cytokines, on photoreceptor protection. We found that exogenous OSM protects both rod and cone photoreceptors. In addition, OSM promotes regeneration of cone outer segments in early stages of cone degeneration. Further investigation showed that OSM treatment induces STAT3 phosphorylation in Müller cells but not in photoreceptors, suggesting that OSM not directly acts on photoreceptors and that the protective effects of OSM on photoreceptors are mediated by Müller cells. These findings support the therapeutic strategy using members of IL-6 family of cytokines for retinal degenerative disorders. They also provide evidence that activation of the STAT3 pathway in Müller cells promotes photoreceptor survival. Our work highlights the importance of Müller cell-photoreceptor interaction in the retina, which may serve as a model of glia-neuron interaction in general.

## Introduction

Retinitis pigmentosa (RP) is a heterogeneous group of inherited retinal degenerative disorders that affect one in 3,500 to 4,000 people [1]. Mutations in 40 genes identified so far are implicated in RP, most of which selectively affect rods (http://www.sph.uth.tmc.edu/retnet/sum-dis.htm). In early stages of RP, patients typically experience night blindness and decline in peripheral vision, due to loss of rod photoreceptors in the peripheral retina. In many cases even though the mutations do not directly affect cone photoreceptors, cones undergo secondary degeneration following rods [1], [2]. The secondary cone degeneration eventually affects the central vision, leading to total blindness. No effective treatments are currently available for RP. How to effectively protect photoreceptors, especially cone photoreceptors, is a major challenge in retinal degeneration research.

Pre-clinical studies have shown that photoreceptors can be protected by exogenous neurotrophic factors. LaVail and colleagues [3] first demonstrated in the RCS (the Royal College of Surgeon) rats that a single injection of FGF-2 successfully rescues photoreceptors from degeneration [3]. A subsequent screen of a panel of factors identified several neurotrophic factors that protect photoreceptors in a light-damaged model, including CNTF (ciliary neurotrophic factor), a member of the interleukin-6 (IL-6) family of cytokines [4]. Many studies since confirmed the protective effects of CNTF in animal models cross several species. These pre-clinical results led to a phase 1 clinical trial with promising results [5]. Two other members of the IL-6 family of cytokines, including CT-1 (cardiotrophin 1) and LIF (leukemia inhibitory factor), have been reported to protect photoreceptors in different animal models [6], [7].

Oncostatin M (OSM), originally isolated from a tumor cell line and identified as a factor to suppress the growth of tumor cells, is also a member of the IL-6 family of cytokines [8], [9], [10]. It expresses mainly in activated T lymphocytes, monocytes [8], [11], but also in other tissues [12], including in neurons, astrocytes, and microglia [13]. OSM has a variety of biological effects, including inhibition of solid tumor cells, inhibition of totipotent embryonic stem cells, and induction of acute phase protein synthesis in hepatic cells [10]. In the nervous system, OSM induces the expression of IL-6 and other cytokines, regulates inflammatory reactions as well as matrix remodeling [13]. It also suppresses brain tumor cells [13]. Elevated levels of OSM have been detected in several human CNS pathologies, such as multiple sclerosis, HIV-associated dementia, and epileptic seizure [14], [15], [16].

In the present work, we studied the neuroprotective feature of OSM in a transgenic rat model of retinal degeneration. Our data show that OSM not only protects both rod and cone photoreceptors, but also promotes regeneration of cone outer segments (COS) in degenerating cones. Furthermore, our results suggest that the effects of OSM are mediated through the Jak/STAT3 pathway in Müller cells.

## Results

### Protection of Rod Photoreceptors by OSM

**Figure 1:**
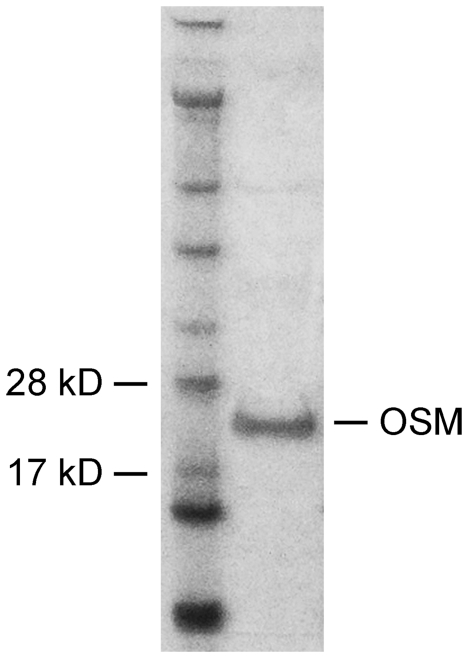
Recombinant human OSM. Purified recombinant human OSM protein was detected as a single band of ∼25 kD after electrophoresis in acrylamide gel and visualized with Coomassie blue. Left lane: protein molecular mass marker (molecular mass is indicated on the left for the two bands near the OSM protein); Right lane: 3 µg of purified recombinant human OSM protein.

Mature human recombinant OSM, fused to a 6xHis tag at the amino terminus, was expressed in E. coli, purified by nickel columns, and buffer exchanged with phosphate buffered saline (PBS) (see Materials and Methods for details). The purified recombinant protein has an apparent size of ∼25 kD after electrophoresis in acrylamide gel (Fig. 1). Although OSM is a glycoprotein, recombinant OSM expressed in E. coli is folded in the native conformation and fully functional as the native protein produced in human cells [17].

**Figure 2:**
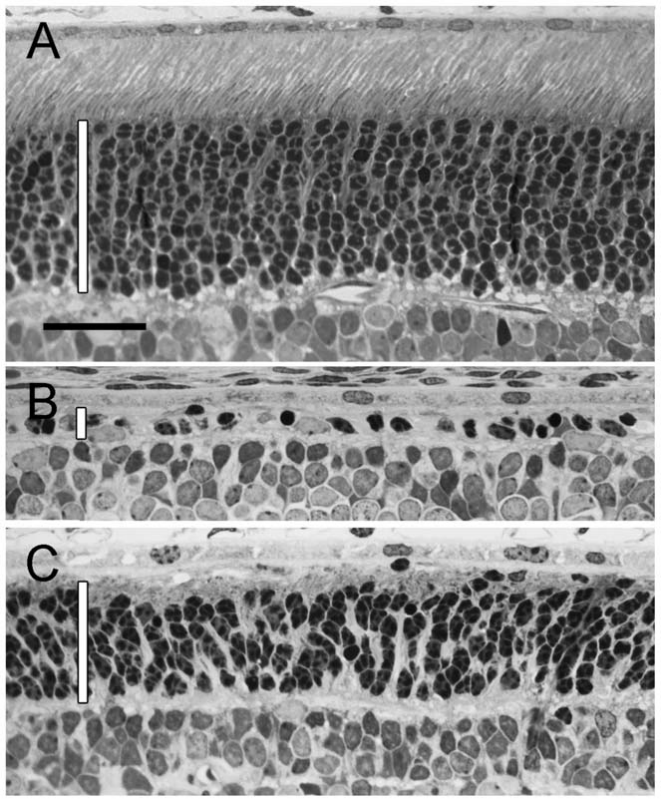
Protection of rod photoreceptors by OSM treatment. Plastic semi-thin sections of retina were examined by light microscopy. In wild-type rats, photoreceptors are well developed by PD20 (A). The ONL contains typically 10–12 rows of nuclei with well-organized outer and inner segments (A). In the retinas of transgenic rats treated with PBS (3 µl, injected at PD9 and harvested at PD 20), the ONL had only one row of nuclei (B). In contrast, more than six rows of nuclei remained in the ONL in OSM-treated eyes (left eyes of the same rats) (C). The ONL is indicated by a white vertical bar in each panel. Scale bar, 25 µm.

To examine the effects of OSM on photoreceptor degeneration, we used the heterozygous S334ter-3 rats as a model of retinal degeneration with rapid degeneration of rod photoreceptors [18]. Intravitreal injection of OSM (10 µg in 3 µl PBS) at PD9 resulted in significant protection of rod photoreceptors when examined at PD20. As shown in Fig. 2, the outer nuclear layer (ONL) of the retina in normal wild type animals has 10–12 rows of photoreceptor nuclei (Fig. 2A), whereas the ONL of S334ter animals treated with PBS had only one row (Fig. 2B). In contrast, OSM-treated retinas typically had 6–8 rows of photoreceptor nuclei in the ONL (Fig. 2C). Measurement of the ONL thickness showed that the thickness in the OSM-treated retinas (23.11±1.70 µm, n = 3) is significantly greater (*P* = 0.002, Student *t* test) than in PBS-treated control retinas (5.22±0.19 µm).

### Protection of Cone Photoreceptors by OSM

**Figure 3:**
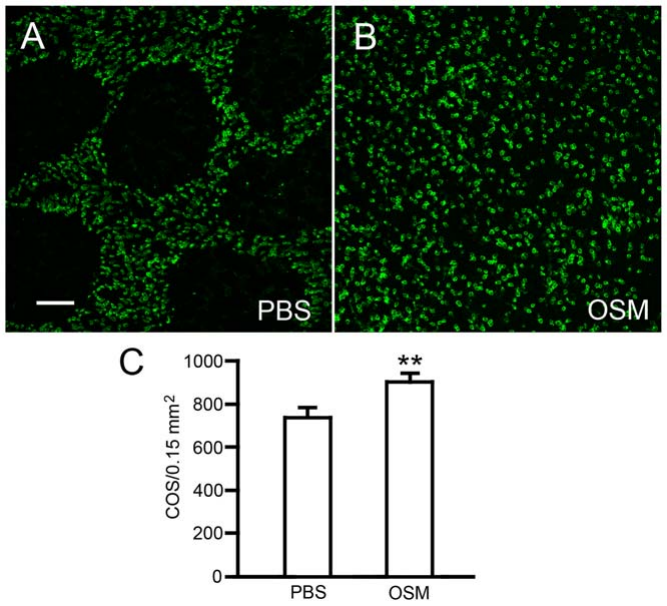
Protection of cone photoreceptors by OSM treatment. Eyes of homozygous S334ter-3 rats were injected with OSM (left eyes) or PBS (right eyes) at PD20 and retinas were collected at PD30. In PBS-treated control eyes, many PNA-negative areas are present (A). In OSM-treated eyes, the PNA-negative areas became very small and completely disappeared (B). Quantitative analysis (C) showed that the density of PNA-positive cells in the OSM-treated retinas (905±35.44, n = 6) is significantly higher than in the PBS-treated retinas (742.83±36.14, n = 6, *P*<0.01, Student *t*-test). Scale bar: 50 µm.

To examine whether OSM protects cone photoreceptors as well, we used the same rat model in which the secondary cone degeneration develops following rod degeneration, as has been characterized recently [19]. In these experiments, eyes of homozygous S334ter rats were injected with OSM (10 µg in 3 µl PBS) at PD 20 and retinas were collected at PD 30. COS were identified by PNA (peanut agglutinin) staining. In the PBS-treated control retinas, loss of COS was found in numerous small PNA-negative areas throughout the retina (Fig. 3A). This finding is consistent with previous observations [19]. In OSM-treated retinas, the PNA-negative areas became very small and in many cases completely disappeared (Fig. 3B). Quantitative analysis showed that the density of PNA-positive cells in the OSM-treated retinas is 905±35.44/0.15 mm^2^ (n = 6), significantly higher than that in the PBS-treated retinas (742.83±36.14, n = 6) (*P*<0.01, Student *t*-test) (Fig. 3C). These results indicate that OSM treatment effectively stops the loss of COS and thus cone degeneration.

### COS Regeneration Induced by OSM Treatment

**Figure 4:**
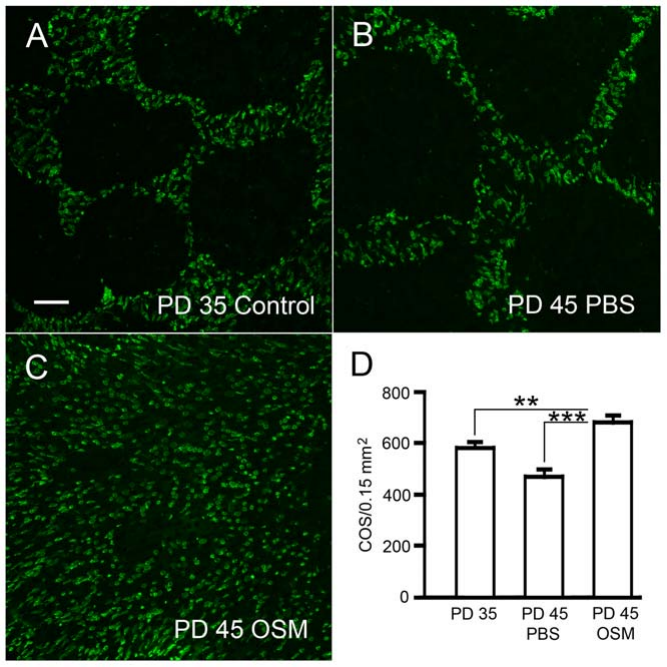
Regeneration of COS induced by OSM. Homozygous S334ter rats were treated with either OSM or PBS at PD35 as described in Fig. 3. Eyes were collected at PD45. A group of eyes was collected at PD35 without treatment as baseline control. There are more PNA positive cells in OSM treated retinas(C) than in either PBS treated retinas (B) or untreated retinas of PD35 (A). Quantitative analysis (D) showed that there are significantly more PNA-positive cells in the OSM-treated retinas at PD45 (665±16.56, n = 3) than in the PBS-treated retinas at PD45 (464±18, n = 3, *P*<0.001) or in untreated retinas at PD35 (565.33±6.36, n = 3, *P*<0.01). Double-asterisk indicates *P*<0.01; triple-asterisk indicates *P*<0.001 (ANOVA and Tukey test). Scale bar: 50 µm.

The disappearance of PNA negative areas in the OSM-treated retinas (Fig. 3B) also suggested to us that OSM promoted regeneration of COS in cones that had lost COS. To test this hypothesis, we performed experiments in which retinas were treated with OSM at PD35 and examined at PD45. As shown in Fig. 4, it appears that there are more PNA-positive cells in OSM-treated retinas at PD45 (Fig. 4C) than in untreated retinas at PD 35 (Fig. 4A) or PBS-treated retinas at PD45 (Fig. 4B). Quantitative analysis showed that indeed, the density of PNA-positive cells in the OSM-treated retinas at PD45 (665±16.56/0.15 mm^2^, n = 3) is significantly higher than that of the untreated retinas collected at PD35 (565.33±6.36, n = 3) (*P*<0.01, ANOVA and Tukey test) or PBS-treated retinas at PD45 (464±18, n = 3) (*P*<0.001, ANOVA and Tukey test) (Fig. 4D). The fact that the density of PNA-positive cells in the OSM-treated retinas at PD45 is higher than that in the retinas at PD35 provides evidence of COS regeneration in response to OSM treatment. This finding is consistent with our previous results with CNTF treatment [19].

### Phosphorylation of STAT3 Induced by OSM

**Figure 5:**
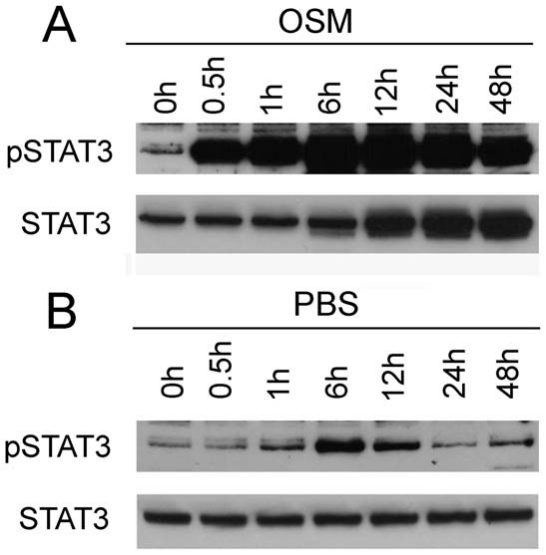
Phosphorylation of STAT3 induced by OSM. Eyes of wild type rats were injected with OSM and retinas were collected at different time points after injection. OSM-induced STAT3 phosphorylation as early as 30 min after treatment (A). OSM treatment also induced an increase in STAT3 protein level in the retina (A). A much smaller increase in STAT3 phosphorylation with shorter duration was seen in retinas treated with PBS (B).

We next examined the phosphorylation of STAT3 in the retina after OSM treatment. A significant increase in STAT3 phosphorylation was detected as early as 30 min after OSM injection (Fig. 5A). The treatment also induced a small increase in STAT3 protein level in the retina (Fig. 5A). In contrast, PBS injection induced a much smaller increase with shorter duration in phosphorylated STAT3 (Fig. 5B), which is consistent with our previous observations [6].

**Figure 6:**
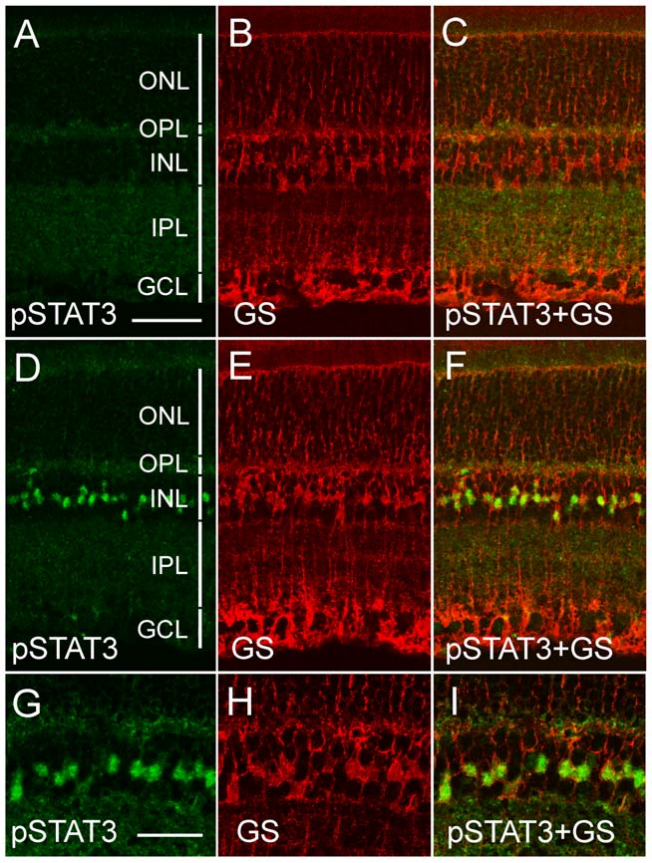
Localization of OSM-induced STAT3 phosphorylation. Retinas (PD20 wild-type rats) were treated with either OSM (left eye) or PBS (right eye) and harvested 1 hr after injection. Sections were double-labeled with anti-pSTAT3 antibodies (pSTAT3, green) and antibodies against glutamine synthetase (GS, red), a Müller cell specific marker. No significant immunoreactivity of pSTAT3 was detected in the control retina (A and C). OSM treatment induced STAT3 phosphorylation is colocalized with GS in a group of cells in the INL (D, F). The colocalization can be clearly seen at a higher magnification (G–I). Layers of retinas were indicated by vertical white bars (A and D). ONL: outer nuclear layer; OPL: outer plexiform layer; INL: inner nuclear layer; IPL: inner plexiform layer, and GCL; ganglion cell layer. Scale bars: 50 µm for A–F; 30 µm for G–I.

To localize OSM-induced STAT3 phosphorylation, wild-type rats were treated with OSM in the left eyes and PBS in the right eyes. Eyes were collected 1 hour later. Phosphorylated STAT3 was detected in the OSM-treated retina in a group of cells in the inner nuclear layer (INL). Those cells were also positive for glutamine synthetase (GS), a Müller cell specific marker, (Fig. 6D–6F, high magnification 6G–6I). The colocalization of pSTAT3 and GS confirms that OSM induces STAT phosphorylation in Müller cells. In contrast, no pSTAT3 was detected in the outer nuclear layer (ONL) where photoreceptor cell bodies reside, indicating that photoreceptors are not directly responsive to OSM (Fig. 6D, 6F). Phosphorylated STAT3 was not detected in the control retina (Fig. 6A). These results provide evidence that OSM induces STAT3 activation specifically in Müller cells, not in photoreceptors.

## Discussion

The present work has shown that OSM protects both rod and cone photoreceptors from degeneration. OSM treatment also promotes regeneration of COS in degenerating cones. We have further demonstrated that OSM induced STAT3 activation in Müller cells, not in photoreceptors, suggesting that OSM-induced photoreceptor survival is mediated by Müller cells.

OSM is a member of the IL-6 family of cytokines with significant homology of amino acid sequence and structure with other members of the family, especially with LIF [9], [10], [20]. As a pleiotrophic cytokine, OSM exerts many functions that overlap those of related cytokines in a variety of systems, including inflammation, cell proliferation, and hematopoiesis, [10], [21], [22]. The expression of OSM is found in various tissues, including the brain [12]. In the brain, OSM expression was reported in hippocampus [23] and pituitary gland [24]. Upregulation of OSM is found in multiple sclerosis lesions in the brain in microglia, reactive astrocytes, infiltrating leukocytes [25], as well as in peripheral blood mononuclear cells [26]. It is also found in interneurons and glial cells in the hippocampus after prolonged seizure [16]. Significant increase in OSM expression was also seen after sciatic nerve crush or transaction [27]. In the OSM-deficient mice, there is a significant decrease in neurons in the dorsal root ganglia [28]. The loss of neurons in the dorsal root ganglia is accompanied with reduced noxious responses to acute thermal, mechanical, chemical, and visceral pain, indicating the essential function of OSM in nociceptor neurons [28]. Furthermore, OSM have been shown to be neuroprotective in models of excitotoxic injury [29], [30]. Our present finding that OSM protects photoreceptors is consistent with OSM being a neurotrophic cytokine, similar to CNTF, LIF, and CT-1.

Two OSM receptor complexes have been identified in human for OSM signal transduction. The type I receptor complex is a heterodimer of LIF receptor β (LIFRβ) and gp130, the same receptor complex shared by several IL-6 family of cytokines, including CNTF, LIF, and CT-1 [31], [32]. The type II OSM receptor complex is composed of the OSM specific receptor OSMRβ and gp130. Human OSM binds to both the type I and type II human receptors [22], [33]. This is not the case in mouse, however. Mouse OSM is unable to transduce signals through mouse LIFRβ. It uses only the receptor complex of mouse OSMRβ and gp130 for its biological activities. On the other hand, human OSM can activate mouse LIFRβ, but not the mouse OSMRβ [33]. In our experiments, we used recombinant human OSM on rat model of retinal degeneration. Although it is not clear whether human OSM interacts with rat OSMRβ, it does induce biological effects on rat cells [34], [35]. It is likely that in rat the human OSM effects are also mediated by the receptor complex of LIFRβ and gp130.

The finding that OSM induces STAT3 phosphorylation in Müller cells but not in photoreceptors is in agreement with previous findings that CNTF and CT-1, two members of the IL-6 family of cytokines, activate the STAT3 pathway in Müller cells but not in photoreceptors [6], [36]. These consistent results strongly suggest that the protective effects of OSM are mediated by Müller cells, which express receptors for cytokines of the IL-6 family [37]. Studies have shown that activation of the STAT3 pathway in Müller cells by CNTF regulates the phototransduction machinery in rod photoreceptors, similar to light-induced photoreceptor plasticity [36], [38]. Together, these results emphasize the importance of Müller cell-photoreceptor interaction in regulating the behavior of photoreceptors. This glia-neuron interaction in the retina may serve as a model of such interaction in other parts of the nervous system.

In summary, our present study demonstrates the neuroprotective effects of OSM on photoreceptor degeneration and suggests the role of Müller cells in mediating the protection. Our work provides additional evidence to support the therapeutic approach of using members of the IL-6 family of cytokines, especially CNTF, for retinal degenerative disorders. Recent results from clinical trials showed that CNTF treatment (CNTF-secreting implants) improves vision and stops the loss of cones in patients with retinal degeneration and geographic atrophy [5], [39], [40]. The clinical results demonstrate that neuroprotection is a viable approach for treating retinal degenerative disorders.

## Materials and Methods

### Ethics Statement

All procedures involving animals adhered to Association for Research in Vision and Ophthalmology Statement for the Use of Animals in Ophthalmic and Vision Research and were approved by the Institutional Animal Care and Use Committee of University of Miami, Miller School of Medicine (07-174).

### Expression and Purification of Human Recombinant OSM Protein

The open reading frame of mature human OSM cDNA was cloned by PCR and subcloned into the expression vector pQE30 (Qiagen, Valencia, CA), fused to a 6xHis tag at the N-terminus to generate plasmid pQE-OSM. Recombinant human OSM protein was expressed in E. coli (XL-blue; Stratagene, La Jolla, CA) and purified by immobilized-metal affinity chromatography on Ni-NTA agarose columns (Qiagen) under native conditions. Eluted protein was buffer-exchanged to PBS, and stored at −80°C in small aliquots. The purified recombinant protein has an apparent size of about 25 kD after electrophoresis (Fig. 1).

### Animals and Intravitreal Injections

Transgenic S334ter-3 rats (Sprague-Dawley background) carrying a murine rhodopsin mutant S334ter [18] and wild-type Sprague-Dawley (Charles River Labs, Wilmington, MA) rats were used in all experiments. Heterozygous S334ter-3 rats were produced by mating homozygous male breeders with wild-type Sprague-Dawley females. Intravitreal injections were delivered through 33-gauge needles connected to 10-µl microsyringes (Hamilton, Reno, NV), as described previously [36]. The left eye of an animal was injected with OSM protein and the right eye with PBS.

### Histology, PNA Staining, and Immunocytochemistry

Animals at a given endpoint were killed by CO_2_ overdose, immediately followed by vascular perfusion with mixed aldehydes [18]. Eyes were embedded in an Epon/Araldite mixture, sectioned at 1 µm thickness to display the entire retina along the vertical meridian. Retina sections were examined by light microscopy.

Cone outer segments were identified by PNA staining, as described previously [19]. Briefly, animals were killed by CO_2_ inhalation and perfused with PBS. Retina-lens preparations were post-fixed in cold 4% paraformaldehyde solution for 4 hours at 4°C. After rinse in PBS, the retina-lens preparations were incubated with Alexa fluor 488-conjugate PNA (Invitrogen, Carlsbad, CA). Retinas were flat-mounted on slides after lenses were removed and examined by confocal microscopy.

For immunostaining, animals were killed by CO_2_ inhalation and perfused with 4% paraformaldehyde. Eyecups were obtained, cryoprotected with 30% sucrose, embedded in the OCT compound (Tissue-Tek; Miles Inc., Elkhart, IN), and frozen in liquid nitrogen. Cryo-sections (12 µm) were incubated with anti-phospho-STAT3 (Tyr705) antibodies (Cell Signaling Technology, Beverly, MA). Immunoreactivity was visualized using the ABC Elite kit (Vector Laboratories, Burlingame, CA) and the Tyramide Signal Amplification Detection System (Invitrogen) according to manufacturers' instructions. Müller cells were identified by antibodies against glutamine synthetase (GS; Millipore Corporation, Temecula, CA). GS immunoreactivity was visualized by Cy3-conjugated secondary antibodies (Jackson Immunoresearch; West Grove, PA). Sections were examined by confocal microscopy.

### Immunoblotting Analysis

Retinas were dissected, snap-frozen in powdered dry ice, and stored at −80°C. Retinas were homogenized and concentration of total protein in each sample was determined by the Bio-Rad protein assay (Bio-Rad Labs, Hercules, CA). Total protein of 20 µg from each sample was electrophoresed in 10% NuPAGE gels (Invitrogen) and transferred to nitrocellular membranes (Bio-Rad Labs). Blots were probed with anti-phosphorylated STAT3 (Tyr705) antibodies (Cell Signaling Technology), visualized using SuperSignal chemiluminescent substrates (Thermo Fisher Scientific) and recorded on Hyperfilm (GE Healthcare, Piscataway, NJ). Blots were then stripped and re-probed with anti-STAT3 antibodies (Cell Signaling Technology).

### Statistical Analysis

Results were analyzed by either Student *t* test or ANOVA followed by Tukey test for comparisons between different groups, using InStat3 (GraphPad Software Inc., San Diego, CA). Data are expressed as mean ± SD.

## References

[1] Hartong DT, Berson EL, Dryja TP (2006). Retinitis pigmentosa.. Lancet.

[2] Delyfer MN, Leveillard T, Mohand-Said S, Hicks D, Picaud S (2004). Inherited retinal degenerations: therapeutic prospects.. Biol Cell.

[3] Faktorovich EG, Steinberg RH, Yasumura D, Matthes MT, LaVail MM (1990). Photoreceptor degeneration in inherited retinal dystrophy delayed by basic fibroblast growth factor.. Nature.

[4] LaVail MM, Unoki K, Yasumura D, Matthes MT, Yancopoulos GD (1992). Multiple growth factors, cytokines, and neurotrophins rescue photoreceptors from the damaging effects of constant light.. Proc Natl Acad Sci U S A.

[5] Sieving PA, Caruso RC, Tao W, Coleman HR, Thompson DJ (2006). Ciliary neurotrophic factor (CNTF) for human retinal degeneration: phase I trial of CNTF delivered by encapsulated cell intraocular implants.. Proc Natl Acad Sci U S A.

[6] Song Y, Zhao L, Tao W, Laties AM, Luo Z (2003). Photoreceptor protection by cardiotrophin-1 in transgenic rats with the rhodopsin mutation s334ter.. Invest Ophthalmol Vis Sci.

[7] Joly S, Lange C, Thiersch M, Samardzija M, Grimm C (2008). Leukemia inhibitory factor extends the lifespan of injured photoreceptors in vivo.. J Neurosci.

[8] Zarling JM, Shoyab M, Marquardt H, Hanson MB, Lioubin MN (1986). Oncostatin M: a growth regulator produced by differentiated histiocytic lymphoma cells.. Proc Natl Acad Sci U S A.

[9] Rose TM, Bruce AG (1991). Oncostatin M is a member of a cytokine family that includes leukemia-inhibitory factor, granulocyte colony-stimulating factor, and interleukin 6.. Proc Natl Acad Sci U S A.

[10] Bruce AG, Linsley PS, Rose TM (1992). Oncostatin M.. Prog Growth Factor Res.

[11] Malik N, Kallestad JC, Gunderson NL, Austin SD, Neubauer MG (1989). Molecular cloning, sequence analysis, and functional expression of a novel growth regulator, oncostatin M.. Mol Cell Biol.

[12] Znoyko I, Sohara N, Spicer SS, Trojanowska M, Reuben A (2005). Comparative studies of oncostatin M expression in the tissues of adult rodents.. Anat Rec A Discov Mol Cell Evol Biol.

[13] Chen SH, Benveniste EN (2004). Oncostatin M: a pleiotropic cytokine in the central nervous system.. Cytokine Growth Factor Rev.

[14] Wallace PM, MacMaster JF, Rouleau KA, Brown TJ, Loy JK (1999). Regulation of inflammatory responses by oncostatin M.. J Immunol.

[15] Ensoli F, Fiorelli V, DeCristofaro M, Santini Muratori D, Novi A (1999). Inflammatory cytokines and HIV-1-associated neurodegeneration: oncostatin-M produced by mononuclear cells from HIV-1-infected individuals induces apoptosis of primary neurons.. J Immunol.

[16] Jankowsky JL, Patterson PH (1999). Differential regulation of cytokine expression following pilocarpine-induced seizure.. Exp Neurol.

[17] Sporeno E, Barbato G, Graziani R, Pucci P, Nitti G (1994). Production and structural characterization of amino terminally histidine tagged human oncostatin M in E. coli.. Cytokine.

[18] Liu C, Li Y, Peng M, Laties AM, Wen R (1999). Activation of caspase-3 in the retina of transgenic rats with the rhodopsin mutation s334ter during photoreceptor degeneration.. J Neurosci.

[19] Li Y, Tao W, Luo L, Huang D, Kauper K (2010). CNTF induces regeneration of cone outer segments in a rat model of retinal degeneration.. PLoS One.

[20] Bruce AG, Hoggatt IH, Rose TM (1992). Oncostatin M is a differentiation factor for myeloid leukemia cells.. J Immunol.

[21] Loy JK, Davidson TJ, Berry KK, Macmaster JF, Danle B (1999). Oncostatin M: development of a pleiotropic cytokine.. Toxicol Pathol.

[22] Grant SL, Begley CG (1999). The oncostatin M signalling pathway: reversing the neoplastic phenotype?. Mol Med Today.

[23] Rosell DR, Nacher J, Akama KT, McEwen BS (2003). Spatiotemporal distribution of gp130 cytokines and their receptors after status epilepticus: comparison with neuronal degeneration and microglial activation.. Neuroscience.

[24] Hanisch A, Dieterich KD, Dietzmann K, Ludecke K, Buchfelder M (2000). Expression of members of the interleukin-6 family of cytokines and their receptors in human pituitary and pituitary adenomas.. J Clin Endocrinol Metab.

[25] Ruprecht K, Kuhlmann T, Seif F, Hummel V, Kruse N (2001). Effects of oncostatin M on human cerebral endothelial cells and expression in inflammatory brain lesions.. J Neuropathol Exp Neurol.

[26] Ensoli F, Fiorelli V, Lugaresi A, Farina D, De Cristofaro M (2002). Lymphomononuclear cells from multiple sclerosis patients spontaneously produce high levels of oncostatin M, tumor necrosis factors alpha and beta, and interferon gamma.. Mult Scler.

[27] Ito Y, Yamamoto M, Li M, Mitsuma N, Tanaka F (2000). Temporal expression of mRNAs for neuropoietic cytokines, interleukin-11 (IL-11), oncostatin M (OSM), cardiotrophin-1 (CT-1) and their receptors (IL-11Ralpha and OSMRbeta) in peripheral nerve injury.. Neurochem Res.

[28] Morikawa Y, Tamura S, Minehata K, Donovan PJ, Miyajima A (2004). Essential function of oncostatin m in nociceptive neurons of dorsal root ganglia.. J Neurosci.

[29] Weiss TW, Samson AL, Niego B, Daniel PB, Medcalf RL (2006). Oncostatin M is a neuroprotective cytokine that inhibits excitotoxic injury in vitro and in vivo.. FASEB J.

[30] Moidunny S, Dias RB, Wesseling E, Sekino Y, Boddeke HW (2010). Interleukin-6-type cytokines in neuroprotection and neuromodulation: oncostatin M, but not leukemia inhibitory factor, requires neuronal adenosine A1 receptor function.. J Neurochem.

[31] Grotzinger J (2002). Molecular mechanisms of cytokine receptor activation.. Biochim Biophys Acta.

[32] Boulanger MJ, Garcia KC (2004). Shared cytokine signaling receptors: structural insights from the gp130 system.. Adv Protein Chem.

[33] Miyajima A, Kinoshita T, Tanaka M, Kamiya A, Mukouyama Y (2000). Role of Oncostatin M in hematopoiesis and liver development.. Cytokine Growth Factor Rev.

[34] Geisterfer M, Richards CD, Gauldie J (1995). Cytokines oncostatin M and interleukin 1 regulate the expression of the IL-6 receptor (gp80, gp130).. Cytokine.

[35] Richards CD, Brown TJ, Shoyab M, Baumann H, Gauldie J (1992). Recombinant oncostatin M stimulates the production of acute phase proteins in HepG2 cells and rat primary hepatocytes in vitro.. J Immunol.

[36] Wen R, Song Y, Kjellstrom S, Tanikawa A, Liu Y (2006). Regulation of rod phototransduction machinery by ciliary neurotrophic factor.. J Neurosci.

[37] Sarup V, Patil K, Sharma SC (2004). Ciliary neurotrophic factor and its receptors are differentially expressed in the optic nerve transected adult rat retina.. Brain Res.

[38] Wen R, Song Y, Liu Y, Li Y, Zhao L (2008). CNTF negatively regulates the phototransduction machinery in rod photoreceptors: implication for light-induced photostasis plasticity.. Adv Exp Med Biol.

[39] Mata NL, Vogel R (2010). Pharmacologic treatment of atrophic age-related macular degeneration.. Curr Opin Ophthalmol.

[40] Talcott KE, Ratnam K, Sundquist SM, Lucero AS, Lujan B (2010). Longitudinal Study of Cone Photoreceptors during Retinal Degeneration and in Response to Ciliary Neurotrophic Factor Treatment.. Invest Ophthalmol Vis Sci [Epub ahead of print].

